# Breastfeeding and dietary habits in childhood and adolescence: the PASOS study

**DOI:** 10.1186/s13006-026-00851-8

**Published:** 2026-05-12

**Authors:** Silvia García, Marina Ródenas-Munar, Luis Carmona-Rosado, Marta Sevilla-Sanchez, Narcis Gusi, Santiago F. Gómez, Marcela González-Gross, Julia Wärnberg, Susana Aznar, Susana Pulgar, Lluís Serra-Majem, Adela Martín-Oliveros, Elena Marín-Cascales, Montse Fitó, Julia Diez, Miguel Ángel González-Valeiro, Jesús Sánchez Gomez, Paula Berruezo, Augusto G. Zapico, Álvaro Oostrom-Rodriguez, Evelyn Martin-Moraleda, Estefanía Herrera-Ramos, Ana Mateos-Lardiés, Pedro E. Alcaraz, Helmut Schröder, Idoia Labayen, Cristina Bouzas, Josep A. Tur

**Affiliations:** 1 Research Group on Community Nutrition and Oxidative Stress, University of Balearic Islands-IUNICS, Palma de Mallorca, E-07122 Spain; 2 Centro de Investigación Biomédica en Red Fisiopatología de la Obesidad y la Nutrición (CIBEROBN), Institute of Health Carlos III, Madrid, 28029 Spain; 3 Health Research Institute of Balearic Islands (IdISBa), Palma, Balearic Islands, E-07120 Spain; 4 Public Health and Epidemiology Research Group, School of Medicine and Health Sciences, Universidad de Alcalá, Alcalá de Henares, Madrid, 28871 Spain; 5 Faculty of Sports Sciences and Physical Education, Universidade da Coruña, A Coruña, Spain; 6 Physical Activity and Quality of Life Research Group (AFYCAV), Faculty of Sport Sciences, University of Extremadura, Cáceres, Spain; 7 Instituto de Investigación e Innovación en el Deporte (INIDE), Cáceres, 10003 Spain; 8 Gasol Foundation Europe, Sant Boi de Llobregat, Barcelona, 08830 Spain; 9 CIBER de Epidemiología y Salud Pública (CIBERESP), Instituto de Salud Carlos III, Madrid, Spain; 10 Cardiovascular Risk and Nutrition Research Group (CARIN), Hospital del Mar Research Institute, Barcelona, Spain; 11 Nursing and Physiotherapy Department, University of Lleida, Lleida, Spain; 12 ImFINE Research Group, Department of Health and Human Performance, Universidad Politécnica de Madrid, Madrid, Spain; 13 EpiPHAAN Research Group, Faculty of Health Sciences, Universidad de Málaga - Instituto de Investigación Biomédica de Málaga (IBIMA Bionand), Málaga, Spain; 14 PAFS Research Group, Faculty of Sports Sciences, University of Castilla-La Mancha- Toledo Campus, Toledo, Spain; 15 Regional Unit of Sports Medicine of Principado de Asturias, Fundación Deportiva Municipal de Avilés, Avilés, Spain; 16 Research Institute of Biomedical and Health Sciences (IUIBS), University of Las Palmas de Gran Canaria, Las Palmas, Spain; 17 Preventive Medicine Service, Centro Hospitalario Universitario Insular Materno Infantil (CHUIMI), Canarian Health Service, Las Palmas, Spain; 18 Sociedad Española de Farmacia Clínica, Familiar y Comunitaria (SEFAC), Madrid, Spain; 19 UCAM Research Center for High Performance Sport, Universidad Católica de Murcia, Murcia, 30107 Spain; 20 Faculty of Sport, Universidad Católica de Murcia, Murcia, 30107 Spain; 21 Cardiovascular Risk and Nutrition Research Group (CARIN), Hospital del Mar Institute for Medical Research, Barcelona, 08003 Spain; 22 ELIKOS Group, Department of Health Sciences, Institute for Sustainability and Food Chain Innovation (IS-FOOD), Public University of Navarre, Pamplona, Spain; 23 Research Group on Community Nutrition and Oxidative Stress, University of the Balearic Islands-IUNICS, IDISBA & CIBEROBN, Guillem Colom Bldg, Campus, Palma de Mallorca, E-07122 Spain

**Keywords:** Breastfeeding, Mediterranean diet, Dietary habits, Children, Adolescents, PASOS study

## Abstract

**Objectives:**

To assess the association between breastfeeding (BF) duration and dietary habits among children and adolescents.

**Design:**

Cross-sectional analyses were conducted using data from two waves of the PASOS study (2019 and 2022), both multicenter observational studies nationally representative.

**Methods:**

A total of 3588 participants from PASOS-2019-2020 and 2809 from PASOS-2022-2023, aged 8-16 years, with sociodemographic, BF, and dietary data were included. BF history was reported by caregivers and categorized into three groups: no BF, BF≤6 months, and BF>6 months. Dietary habits were assessed through a Mediterranean diet (MD) adherence validated index: the KIDMED. Associations between BF duration and KIDMED items were assessed using ANOVA, Chi-squared tests, and logistic regression models.

**Results:**

Longer BF duration was associated with higher adherence to MD in both surveys. In 2019-2020, mean (SD) KIDMED scores were: no BF 6.5 (2.4), BF≤6 months 6.7 (2.4), and BF >6 months 7.0 (2.4), (p<0.001); in 2022-2023, corresponding values were 6.2 (2.4), 6.4 (2.5), and 6.7 (2.4), (p<0.001). Logistic regression analyses showed that in both 2019-2020 and 2022-2023, BF>6 months was associated with lower odds of skipping breakfast and higher odds of consuming cereals at breakfast, vegetables, and rice or pasta. Several associations remained significant after adjustment for exclusive BF and parents’ education, employment status, and age, although effect sizes were attenuated.

**Conclusions:**

Longer BF duration was associated with healthier dietary habits and higher adherence to the MD in children and adolescents, but these associations may reflect broader early-life family and socio-educational contexts rather than a direct causal effect of BF itself.

**Clinical trial registration:**

The trial was officially recorded in 2019 with the International Standard Randomized Controlled Trial (ISRCT), bearing the identification number 34,251,612 accessed on 08/08/2019 https//www.isrctn.com/ISRCTN34251612 (21).

## Introduction

The way an infant is fed during its first stages of life is crucial. Breastfeeding (BF) can be called the gold standard for infant feeding. Breast milk is recognized as the optimal source of nutrition for infants, offering a safe and hygienic option that supplies immunological components to protect against common childhood infections. It fully meets infants’ energy and nutrient requirements during the initial months of life and remains a key contributor, thereafter, providing up to half or more nutritional needs in the second half of the first year and about one third throughout the second year. However, in many Western countries, the average duration of BF is relatively short, and often limiting the extent to which these nutritional contributions are achieved in practice [1].

In addition to covering nutritional requirements, BF has started to be studied for its possible long-term beneficial effects [2]. A BF period of 6–12 months may be related to enhanced behavioral developmental and language outcomes [3]. A recent review also pointed out that BF exerts a modest positive influence on later childhood intelligence [4], a fact that was supported by the World Health Organization (WHO) in a 2013 systematic review assessing the long-term effects of BF [5]. However, other studies have reported no significant associations between BF and neurocognitive development or academic performance in later childhood [6, 7]. This WHO systematic review also found a substantial protective effect against overweight, obesity and type 2 diabetes related to BF;^5^ associations that have been strengthened in recent studies [8, 9]. BF was also studied as a therapeutic option, such as serving as the best source of intact protein for infants under 6 months with hyperphenylalaninemia or phenylketonuria, ensuring adequate nutrition and metabolic control [10]. Beyond the documented benefits for children, BF also confers health advantages for mothers; recent studies suggested that ever BF or longer BF duration may be associated with low risks of breast cancer, epithelial ovarian cancer, hypertension, and type 2 diabetes in mothers [1, 9, 11]. These maternal benefits have been observed across a range of BF durations and are not restricted to the first six months [9, 12]. Both parental educational level and targeted interventions aimed at supporting BF could enhance BF practices, and, in fact broader nutritional interventions have been associated with improvements in feeding practices during childhood in some studies, and positively influenced children’s diet and overall well-being [13, 14].

In light of these recognized benefits, international health authorities have established clear recommendations. WHO and UNICEF advised that BF should be initiated within the first hour after birth and maintained exclusively for the first six months of life, with no additional foods or liquids, not even water. Infants should be breastfed on demand, both during the day and night, and the use of bottles, teats, or pacifiers is discouraged. From six months onward, nutritionally adequate and safe complementary foods should be introduced, while BF continues up to two years of age or beyond [1]. Despite this guidance and the strong evidence supporting the maternal and child health benefits of BF, many mothers still did not breastfeed their children or breastfeed for a shorter duration than recommended, for a variety of reasons [15, 16]. The fact is the specific benefits associated with different BF durations remain unclear, as the optimal duration of BF and the extent to which longer BF confers additional advantages have yet to be clearly defined. Although the benefits of exclusive BF during the first six months of life are widely accepted [17], the evidence supporting additional benefits of prolonged BF beyond the first months or up to two years of age is less clearly established in the literature. An increasing number of studies have begun to examine whether different BF durations are associated with health, developmental, or behavioral outcomes during school age and later stages of life [18, 19]; nevertheless, the findings of these studies differ, with some reporting small favorable associations and others finding no clear relationship. This heterogeneity partly reflects differences in study design and methodological quality, as many observational studies vary in their ability to adequately control for socioeconomic, educational, and environmental confounders [9, 20, 21]. In addition, reverse causation has been highlighted as an important source of bias in BF research, as mothers may discontinue BF earlier when infants appear healthy and growing well, and continue when concerns arise. Studies that do not account for this dynamic may misinterpret the direction of the association [20, 22]. In this context, it is important not to forget that family-level, socio-educational, and dietary environmental factors may represent key elements in this research area and should be carefully considered when interpreting associations between BF duration and later child outcomes, as these factors may influence both infant feeding practices and subsequent health-related behaviors.

To date, research has primarily concentrated on cognitive and physiological outcomes of BF, while the potential association with children’s dietary habits and specific diet has received far less attention. In our context, dietary patterns are commonly assessed through the adherence to the Mediterranean Diet (MD), which serves as a benchmark for evaluating overall diet quality and healthy eating behaviors. In this regard, the MD provides a well-established framework for assessing dietary patterns. The MD is widely recognized as one of the healthiest dietary models. Adherence to the MD during childhood and adolescence is associated with improved cardiometabolic profiles, better weight management, and a reduced risk of obesity and related disorders [23, 24]. Although traditionally rooted in Mediterranean regions, the MD has been adopted as a reference model for healthy eating, while also reflecting cultural heritage and evidence-based nutritional quality [25, 26].

Given the role of early-life exposures in shaping later health-related behaviors, BF duration may be associated with the development of dietary patterns and lifestyle habits during childhood and adolescence. However, despite the well-established benefits of BF, the potential link between BF duration and later dietary quality remains insufficiently explored, and it is still unclear whether different BF durations confer differential benefits in these domains. Moreover, the mechanisms underlying these associations may involve both biological pathways and social/behavioral factors, and our study considers both perspectives when formulating its hypothesis. In this context, we hypothesized that a longer duration of BF would be associated with greater adherence to Mediterranean dietary habits and healthier dietary behaviors during childhood and adolescence. Therefore, the aim of this study was to examine the association between BF duration and adherence to the MD and dietary habits among children and adolescents, as well as their parents.

## Methods

### Study design

The present work is based on two cross-sectional analyses within the frame of the Physical Activity, Sedentarism, and Obesity in Spanish Youth study (PASOS). Data from the first edition (PASOS-2019) and the second edition (PASOS-2022), both multicenter, observational studies nationally representative, were used and compared. The PASOS study protocol was previously described and published [27].

### Participants, recruitment and ethics

The first edition of the Physical Activity, Sedentarism, and Obesity in Spanish Youth study (PASOS-2019-2020) was conducted between March 2019 and February 2020. The participants in this study were Spanish children and adolescents of both sexes aged 8 to 16 years. A total of 3802 participants were recruited through 247 educational centers proportionally selected across the 17 autonomous communities of Spain. In this edition, sociodemographic, BF, and dietary data was available for 3588 participants.

The second edition (PASOS-2022-2023) followed the same methodology and inclusion criteria. Written informed consent was obtained from parents or legal guardians before enrolment of each child, and they also signed their own written consent. Data of 3176 participants was collected from March 2022 to June 2023, but just 2809 had available sociodemographic, BF, and dietary data. The participants in this study were also Spanish children and adolescents of both sexes aged 8 to 16 years.

In both editions, Data was collected by trained researchers from 14 research groups comprising more than 70 researchers each. Participants were children or adolescents aged 8 to 16 and living in Spain. Students with intellectual disabilities that prevented them from completing the questionnaires were not eligible to participate. Each potential exclusion was assessed by both teachers and parents/legal guardians prior to confirmation. An additional filter was applied whereby individuals identifying as non‑binary were excluded, as this allowed the sample to be stratified using a dichotomous sex variable for the analyses. In accordance with the principles of the Declaration of Helsinki, the PASOS study received approval from the Ethics Committee of the Fundació Sant Joan de Déu, Barcelona, Spain (ref. PIC-179-18; 17 December 2018). The trial was officially recorded in 2019 with the International Standard Randomized Controlled Trial (ISRCT), bearing the identification number 34,251,612; accessed on 08 August, 2019; https://www.isrctn.com/ISRCTN34251612 [28].

### Data collection

All questionnaires were self-reported using online software and completed by the participants in the presence of a field researcher previously trained by the Gasol Foundation. To ensure consistency and minimize variability across observers, all researchers participated in a one-day training session on the project methodology organized by the Gasol Foundation.

Data collection was conducted through two main sources:

- Validated questionnaires administered to children and adolescents to gather information on lifestyle variables, and to parents/legal guardians to obtain data on lifestyle behaviors, socio-economic factors, and environmental variables.
- Anthropometric measurements, including weight, height, and waist circumference of the participating children and adolescents.

### Breastfeeding (BF) data

Information about BF was covert, asking three questions: Was your child breastfed? For how many weeks did you breastfeed your child? How many weeks did you breastfeed your child exclusively, without other liquids (formula, water, juices, etc.) or solids?

Questions were asked separately to both parents which were codified in adult 1 and adult 2. Adult 1 was considered as the person taking care of the child most part of the time which answers were used for the current study. The number of weeks was converted into months to allow better understanding of data.

**Fig. 1:**
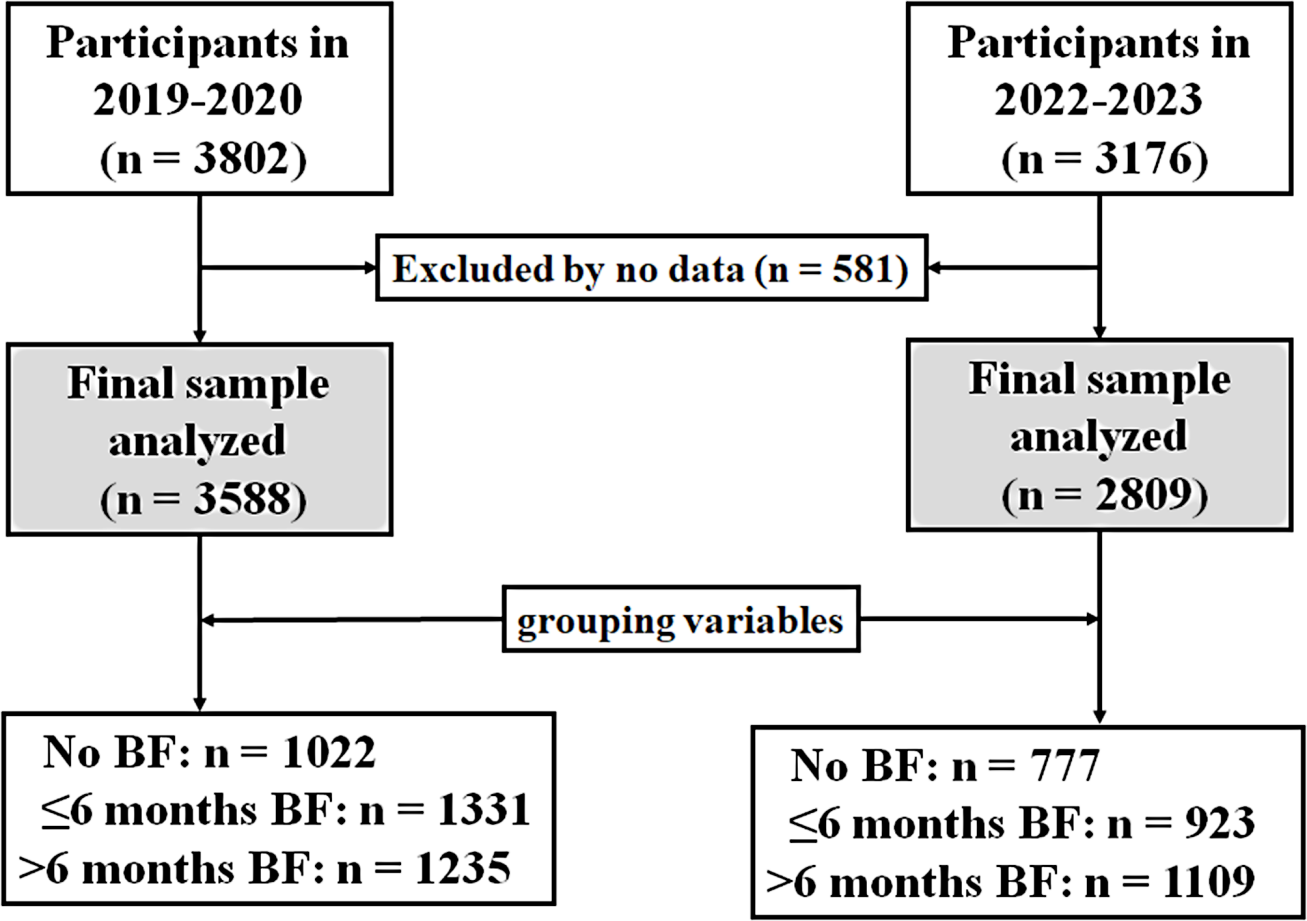
Flow-chart of the sample. Abbreviations: BF; Breastfeeding

The total sample of each separated edition was divided into three groups according to the total BF duration, determined in months: No BF, ≤ 6 months of BF, and > 6 months of B (see Fig. 1). The cut-off point was based on the WHO recommendation of a minimum exclusive BF duration of six months [1].

### Eating habits and mediterranean diet adherence

The 16-item KIDMED index questionnaire was used to determine children and adolescents eating habits, and their MD adherence. The KIDMED is a tool specifically developed and validated in Spanish children and adolescents [29–31]. It consists of 16 dichotomous items that cover different aspects related to breakfast quality, frequency of consumption of key food groups, and the intake of fast food and other low-quality dietary patterns. Items reflecting a negative connotation towards adherence to the MD were scored as -1, while those with a positive connotation were scored as + 1. The total score ranged from − 4 to 12 points, and adherence levels were categorized as low (-4 to 3), medium (4 to 7), and high (8 to 12). Our primary outcomes were the KIDMED total score and adherence category. Item-level analyses were considered exploratory. Given the large number of comparisons, we interpreted item-level results cautiously, focusing on effect sizes and confidence intervals (CIs) rather than statistical significance.

### Statistics

Statistical analysis was performed with the Statistical Package for the Social Sciences version 27.0 (IBM SPSS Statistics for Windows, Version 27.0. Armonk, NY, USA: IBM Corp). Frequency (n) and percentage (%) were presented for qualitative variables; descriptive statistics for quantitative variables were presented as mean and standard Deviation (SD) (Tables 1 and 2).

One-way ANOVA with Bonferroni’s post-hoc test was used for continuous variables, with different superscript letters (a, b, c) show statistically significant differences between groups. The Chi-squared test (χ^2^) was used for categorical variables. Statistical significance was determined at *p* < 0.05 (Table 3). The association between the BF groups and KIDMED items was analyzed using a binomial logistic regression, G1 was considered as the reference. Odds Ratio (OR) value, crude and adjusted (by exclusive BF and by parent’s education, employment status and age), and its 95% CIs were calculated. The p-value of the total association was shown, and the different group p-values were marked as *p < 0.05 and **p < 0.01 (Tables 4 and 5). Binomial logistic regression models were also used to estimate OR (crude and adjusted by exclusive BF) and 95% CIs for the association between primary caregiver sociodemographic characteristics and children/adolescents’ anthropometric variables with breastfeeding history (Table 6).

## Results

### Sociodemographic characteristics of caregivers and participants

Tables 1 and 2 show sociodemographic characteristics of caregivers and participants and BF descriptive statistics. In 2019–2020, a total of 3588 caregiver-child pairs were analyzed, whereas in 2022–2023 the sample comprised 2809 pairs.

Most primary caregivers were female, and the proportion increased from 77.6% in 2019–2020 to 85.0% in 2022–2023. Parent’s education differed significantly between both studies, with a higher proportion of university-educated caregivers in 2022–2023 (36.3%) compared with 2019–2020 (29.2%). Most caregivers were employed in both studies, with similar distributions of job status and mean age; 44.5 years in 2019 vs. 44.7 in 2022. Average BF duration increased from 6.2 months (SD 9.7) in 2019 to 6.7 months (SD 10.6) in 2022, as did exclusive BF (3.6 vs. 3.9 months).

Regarding children and adolescents, sex distribution and education was balanced across years. The mean age was 12.5 years old in both cohorts, with similar mean weight, height and BMI z-scores.

**Table 1:** Infant feeding practices and sociodemographic characteristics of the primary caregiver in 2019–2020 and 2022–2023

	2019–2020 (*n* = 3588)	2022–2023 (*n* = 2809)
Primary caregiver age (years old) #	44.5 (6.1)	44.7 (6.0)
Sex (female) ^	2783 (77.6)	2389 (85.0)
Education ^		
University	1046 (29.2)	1019 (36.3)
Secondary	1957 (54.5)	1427 (50.9)
Primary	471 (13.1)	292 (10.4)
No Education	53 (1.5)	18 (0.6)
Didn’t respond	61 (1.7)	53 (1.9)
Employment status ^		
Working	2688 (74.9)	2156 (76. 8)
Household tasks	449 (12.5)	330 (11.7)
Unemployment	296 (8.2)	195 (6.9)
Student	32 (0.9)	27 (1.0)
Retirement	21 (0.6)	16 (0.6)
Permanent disability	46 (1.3)	33 (1.2)
Didn’t respond	56 (1.6)	52 (1.9)
Months of BF #	6.2 (9.7)	7.4 (10.9)
Exclusive months of BF #	3.6 (2.8)	3.9 (2.8)

Abbreviations: BF; Breastfeeding. SD; Standard Deviation. #mean (SD); ^n (%)

**Table 2:** Sociodemographic and dietary characteristics of children and adolescents in 2019 and 2022

	2019–2020 (*n* = 3588)	2022–2023 (*n* = 2809)
Age (years old) #	12.5 (2.3)	12.5 (2.3)
Sex (female) ^	1850 (51.6)	1424 (50.7)
Education ^		
Primary	1802 (50.2)	1395 (49.7)
Secondary	1786 (49.8)	1414 (50.3)
Weight (kg) #	48.7 (15.1)	48.6 (15.1)
Height (cm) #	153.1 (13.5)	154.0 (14.0)
zBMIscore #	0.5 (1.1)	0.4 (1.2)
KIDMED index score #	6.7 (2.4)	6.5 (2.5)

Abbreviations: BMI; Body Mass Index. SD; Standard Deviation. #mean (SD); ^n (%)

### Mediterranean diet adherence and breastfeeding history

Table 3 shows differences in KIDMED index scores and MD adherence levels according to children’s and adolescents’ BF history in 2019–2020 and 2022–2023, respectively. In both cohorts, longer BF duration was associated with higher KIDMED scores. In 2019–2020, children breastfed for more than 6 months had higher adherence (mean score 7.0) compared with those breastfed for ≤ 6 months (6.7) or not breastfed (6.5; *p* < 0.001). Similar trends were observed in 2022 (6.7, 6.4, and 6.2, respectively; *p* < 0.001). MD adherence levels by BF duration were also significant in both years. Consistently, the proportion of children with high adherence increased notably with BF duration and was the highest in breastfed children for more than 6 months. A higher prevalence of low adherence was seen in the group with no BF time in both studies. The distribution of medium adherence varied between survey years and did not follow a consistent pattern. Although these patterns suggest that children breastfed for longer periods tended to show healthier dietary profiles, these associations should be interpreted cautiously, as they may partly reflect underlying family, socio‑educational, or cultural differences associated with BF practices.

**Table 3:** KIDMED index score and MD adherence level according to children/adolescent’s breastfeeding history in 2019 and 2022

	NO BF	≤ 6 months BF	> 6 months BF	*p*-value
Participants ^				< 0.001
2019–2020	1022 (28.4)	1331 (37.1)	1235 (34.5)	
2022–2023	777 (27.7)	923 (32.8)	1109 (39.5)	
KIDMED index score #				
2019–2020	6.5 (2.4) ^b^	6.7 (2.4) ^c^	7.0 (2.4) ^b c^	< 0.001
2022–2023	6.2 (2.5) ^b^	6.4 (2.5) ^c^	6.7 (2.4) ^b c^	< 0.001
MD adherence level ^				
2019–2020				
Low	176 (17.2)	180 (13.5)	171 (13.8)	< 0.001
Medium	492 (48.1)	655 (49.2)	536 (43.4)	
High	354 (34.6)	496 (37.3)	528 (42.8)	
2022–2023				
Low level	115 (14.8)	124 (13.5)	117 (10.5)	0.006
Medium level	404 (52.0)	466 (50.5)	542 (48.9)	
High level	258 (33.2)	333 (36.1)	450 (40.6)	

Abbreviations: BF; Breastfeeding. KIDMED; Mediterranean diet questionnaire for children and adolescents. MD; Mediterranean diet; SD: Standard Deviation. Differences in means between groups were tested using one-way ANOVA and Bonferroni post-hoc: superscript letters show statistically significant differences between groups. Differences in prevalence’s across groups were examined using χ^2^. #mean (SD); ^n (%)

Logistic regression analyses exploring the association between BF duration and individual dietary behaviors assessed by the KIDMED questionnaire are presented in Table 4 (2019–2020) and Table 5 (2022–2023). These item-level analyses should be interpreted as exploratory, with emphasis placed on the magnitude and direction of the effect estimates rather than isolated p-values.

**Table 4:** Odds ratio (and 95% confidence interval) from logistic regression showing the association of children/adolescent’s “yes” answers in the KIDMED questionnaire with children/adolescent’s breastfeeding history in 2019–2020

			NO BF (*n* = 1022)			≤ 6 months BF (*n* = 1331)	> 6 months BF (*n* = 1235)	*p-value*
		OR (95% CI)						
Q1. Skips breakfast (−)	*Crude OR*			Ref. (1.00).	0.81 (0.64–1.05)		0.66 (0.51–0.86) **	0.008
	*OR adjusted 1*				0.87 (0.41–1.82)		0.52 (0.25–1.10)	0.015
Q2. Takes dairy product for breakfast (+)	*Crude OR*			Ref. (1.00).	1.17 (0.95–1.44)		1.35 (1.09–1.68) **	0.027
	*OR adjusted 1*				1.05 (0.55–2.03)		1.42 (0.73–2.76)	0.126
Q3. Takes cereal or grains product for breakfast (+)	*Crude OR*			Ref. (1.00).	1.13 (0.95–1.31)		1.35 (1.13–1.61) **	0.004
	*OR adjusted 1*				1.15 (0.68–1.96)		1.43 (0.83–2.44)	0.124
Q4. Takes pastries/commercially baked goods for breakfast (−)	*Crude OR*			Ref. (1.00).	0.98 (0.82–1.18)		0.94 (0.79–1.13)	0.800
	*OR adjusted 1*				0.84 (0.48–1.46)		0.73 (0.42–1.28)	0.356
Q5. Takes a fruit or fruit juice daily (+)	*Crude OR*			Ref. (1.00).	1.11 (0.92–1.34)		1.09 (0.91–1.32)	0.492
	*OR adjusted 1*				1.09 (0.62–1.91)		1.14 (0.65–2.01)	0.866
Q6. Takes a second serving of fruit daily (+)	*Crude OR*			Ref. (1.00).	1.09 (0.93–1.30)		1.23 (1.04–1.46) *	0.055
	*OR adjusted 1*				0.98 (0.58–1.66)		0.99 (0.58–1.69)	0.991
Q7. Consumes yogurts and/or 40 g cheese daily (+)	*Crude OR*			Ref. (1.00).	1.24 (1.01–1.51) *		1.21 (0.99–1.48)	0.073
	*OR adjusted 1*				0.87 (0.46–1.67)		0.83 (0.43–1.60)	0.828
Q8. Consumes raw or cooked vegetables daily (+)	*Crude OR*			Ref. (1.00).	1.08 (0.91–1.29)		1.42 (1.18–1.69) **	< 0.001
	*OR adjusted 1*				0.52 (0.29–0.96) *		0.63 (0.34–1.16)	0.053
Q9. Consumes raw or cooked vegetables more than 1/day (+)	*Crude OR*			Ref. (1.00).	0.94 (0.78–1.13)		1.22 (1.03–1.47) *	0.006
	*OR adjusted 1*				0.41 (0.24–0.71) **		0.52 (0.30–0.90) *	0.002
Q10. Regular fish consumption (at least 2–3/week) (+)	*Crude OR*			Ref. (1.00).	1.08 (0.91–1.28)		0.94 (0.79–1.12)	0.261
	*OR adjusted 1*				0.80 (0.46–1.39)		0.70 (0.40–1.22)	0.288
Q11. Goes > 1/week fast food restaurant (−)	*Crude OR*			Ref. (1.00).	0.94 (0.78–1.15)		0.81 (0.66–0.99) *	0.112
	*OR adjusted 1*				1.80 (0.88–3.66)		1.46 (0.71–2.99)	0.126
Q12. Regular nut consumption (≥ 2–3/week) (+)	*Crude OR*			Ref. (1.00).	0.93 (0.79–1.10)		1.09 (0.92–1.29)	0.182
	*OR adjusted 1*				1.03 (0.61–1.75)		1.07 (0.63–1.83)	0.915
Q13. Likes pulses and eats more than 1/week (+)	*Crude OR*			Ref. (1.00).	1.24 (1.04–1.49) *		1.11 (0.92–1.33)	0.063
	*OR adjusted 1*				1.26 (0.72–2.20)		1.08 (0.61–1.91)	0.407
Q14. Takes sweets and candies several times every day (−)	*Crude OR*			Ref. (1.00).	1.08 (0.89–1.32)		0.93 (0.75–1.14)	0.289
	*OR adjusted 1*				1.21 (0.64–2.30)		0.98 (0.51–1.89)	0.331
Q15. Consumes rice or pasta almost daily (≥5/week) (+)	*Crude OR*			Ref. (1.00).	0.88 (0.74–1.04)		1.09 (0.92–1.30)	0.022
	*OR adjusted 1*				0.74 (0.44–1.25)		0.86 (0.50–1.45)	0.294
Q16. Uses of olive oil at home (+)	*Crude OR*			Ref. (1.00).	1.25 (0.94–1.68)		1.21 (0.90–1.63)	0.265
	*OR adjusted 1*				1.96 (0.90–4.27)		1.76 (0.80–3.86)	0.235

Abbreviations: BF; Breastfeeding. Q; Question. OR; Binomial logistic regression. OR 1 was adjusted by exclusive breastfeeding duration and parent’s education, employment status and age. Separated quartile p-values: *p < 0.05 and **p < 0.01

**Table 5:** Odds ratio (and 95% confidence interval) from logistic regression showing the association of children/adolescent’s “yes” answers in the KIDMED questionnaire with children/adolescent’s breastfeeding history in 2022–2023

			NO BF (*n* = 777)	≤ 6 months BF (*n* = 923)		> 6 months BF (*n* = 1109)	*p-value*
		OR (95% CI)					
Q1. Skips breakfast (−)	*Crude OR*		Ref. (1.00).		1.11 (0.86–1.42)	0.83 (0.64–1.06)	0.047
	*OR adjusted 1*				0.85 (0.37–1.93)	0.65 (0.29–1.48)	0.224
Q2. Takes dairy product for breakfast (+)	*Crude OR*		Ref. (1.00).		0.96 (0.77–1.21)	1.04 (0.84–1.30)	0.784
	*OR adjusted 1*				1.57 (0.76–3.24)	1.75 (0.85–3.60)	0.278
Q3. Takes cereal or grains product for breakfast (+)	*Crude OR*		Ref. (1.00).		1.01 (0.83–1.22)	1.35 (1.12–1.63) **	< 0.001
	*OR adjusted 1*				0.48 (0.23-1.00)	0.62 (0.30–1.27)	0.040
Q4. Takes pastries/commercially baked goods for breakfast (−)	*Crude OR*		Ref. (1.00).		0.98 (0.80–1.21)	0.94 (0.77–1.15)	0.830
	*OR adjusted 1*				1.12 (0.53–2.36)	1.09 (0.52–2.28)	0.941
Q5. Takes a fruit or fruit juice daily (+)	*Crude OR*		Ref. (1.00).		1.02 (0.84–1.25)	1.17 (0.96–1.42)	0.220
	*OR adjusted 1*				1.79 (0.90–3.56)	1.87 (0.95–3.70)	0.196
Q6. Takes a second serving of fruit daily (+)	*Crude OR*		Ref. (1.00).		0.97 (0.80–1.17)	1.22 (1.01–1.46) *	0.021
	*OR adjusted 1*				0.41 (0.20–0.85) *	0.52 (0.26–1.08)	0.017
Q7. Consumes yogurts and/or 40 g cheese daily (+)	*Crude OR*		Ref. (1.00).		1.15 (0.91–1.46)	1.09 (0.87–1.37)	0.489
	*OR adjusted 1*				2.10 (0.99–4.47)	1.92 (0.91–4.04)	0.153
Q8. Consumes raw or cooked vegetables daily (+)	*Crude OR*		Ref. (1.00).		1.21 (0.99–1.47)	1.26 (1.04–1.52) *	0.048
	*OR adjusted 1*				0.69 (0.31–1.55)	0.61 (0.27–1.37)	0.371
Q9. Consumes raw or cooked vegetables more than 1/day (+)	*Crude OR*		Ref. (1.00).		1.09 (0.89–1.34)	1.23 (1.02–1.50) *	0.095
	*OR adjusted 1*				0.78 (0.39–1.57)	0.85 (0.43–1.70)	0.670
Q10. Regular fish consumption (at least 2–3/week) (+)	*Crude OR*		Ref. (1.00).		1.25 (1.03–1.52) *	1.36 (1.13–1.64) **	0.004
	*OR adjusted 1*				2.03 (1.02–4.07) *	1.93 (0.97–3.86)	0.135
Q11. Goes > 1/week fast food restaurant (−)	*Crude OR*		Ref. (1.00).		0.83 (0.66–1.04)	0.80 (0.64–0.99) *	0.115
	*OR adjusted 1*				0.28 (0.14–0.57) **	0.35 (0.17–0.70) **	0.002
Q12. Regular nut consumption (≥ 2–3/week) (+)	*Crude OR*		Ref. (1.00).		0.96 (0.79–1.16)	1.15 (0.95–1.38)	0.097
	*OR adjusted 1*				0.79 (0.40–1.56)	0.92 (0.47–1.80)	0.438
Q13. Likes pulses and eats more than 1/week (+)	*Crude OR*		Ref. (1.00).		1.15 (0.93–1.42)	1.11 (0.90–1.35)	0.426
	*OR adjusted 1*				0.38 (0.14–1.03)	0.37 (0.14-1.00) *	0.148
Q14. Takes sweets and candies several times every day (−)	*Crude OR*		Ref. (1.00).		0.89 (0.71–1.11)	0.82 (0.66–1.03)	0.218
	*OR adjusted 1*				0.47 (0.22–0.97) *	0.54 (0.26–1.11)	0.111
Q15. Consumes rice or pasta almost daily (≥5/week) (+)	*Crude OR*		Ref. (1.00).		0.78 (0.64–0.95) *	0.85 (0.70–1.02)	0.038
	*OR adjusted 1*				0.36 (0.17–0.74) **	0.38 (0.18–0.78) **	0.021
Q16. Uses of olive oil at home (+)	*Crude OR*		Ref. (1.00).		0.91 (0.66–1.24)	0.98 (0.72–1.33)	0.800
	*OR adjusted 1*				1.03 (0.29–3.57)	0.89 (0.26–3.10)	0.783

Abbreviations: BF; Breastfeeding. Q; Question. OR; Binomial logistic regression. OR 1 was adjusted by exclusive breastfeeding duration and parent’s education, employment status and age. Separated quartile p-values: *p < 0.05 and **p < 0.01

In the 2019–2020 cohort, children breastfed for more than six months generally showed lower odds of less healthy behaviors and higher odds of several MD compatible habits compared with non-breastfed children. In crude models, longer BF was associated with reduced odds of skipping breakfast (OR = 0.66) and greater likelihood of consuming cereals or grains at breakfast (OR = 1.35), vegetables daily (OR = 1.42), and vegetables more than once per day (OR = 1.22). Some additional tendencies were observed for higher consumption of dairy products at breakfast and legumes. However, after adjustment for exclusive BF duration and parental sociodemographic characteristics, most associations were attenuated and CIs widened, although the direction of the associations generally remained similar.

A comparable pattern was observed in the 2022–2023 cohort. Children breastfed for more than six months tended to show higher odds of several favorable dietary behaviors, including consumption of cereals at breakfast (OR = 1.35), a second daily serving of fruit (OR = 1.22), vegetables (OR = 1.26), and regular fish consumption (OR = 1.36) in crude analyses. They also tended to show lower odds of frequent fast-food consumption. After adjustment, the magnitude of several estimates was reduced and CIs often included the null value, although the overall direction of the associations remained broadly consistent with the crude analyses.

**Table 6:** Odds ratio (and 95% confidence interval) from logistic regression showing the association of sociodemographic characteristics of the primary caregiver and the anthropometric characteristics of children/adolescents with children/adolescent’s breastfeeding history across the two study periods (2019–2020 and 2022–2023)

2019–2020		No BF (*n* = 1022)			≤ 6 months BF (*n* = 1331)	> 6 months BF (*n* = 1235)	*p*-value
Primary caregiver age	Crude OR		Ref. (1.00).	1.02 (0.86–1.20)		0.87 (0.74–1.03)	0.110
	OR adjusted 1			1.56 (0.91–2.65)		1.16 (0.67–1.98)	0.012
Primary caregiver education (university or not)	Crude OR		Ref. (1.00).	0.48 (0.40–0.57) **		0.40 (0.33–0.48) **	< 0.001
	OR adjusted 1			0.37 (0.19–0.74) **		0.32 (0.16–0.64) **	0.004
Primary caregiver employment status (working or not)	Crude OR		Ref. (1.00).	0.35 (0.30–0.42) **		0.40 (0.33–0.47) **	< 0.001
	OR adjusted 1			0.55 (0.32–0.96) *		0.62 (0.36–1.08)	0.096
Child’s Weight (kg)	Crude OR		Ref. (1.00).	1.16 (0.99–1.36)		0.97 (0.83–1.14)	0.052
	OR adjusted 1			1.65 (0.98–2.80)		1.45 (0.85–2.45)	0.116
Child’s Height (cm)	Crude OR		Ref. (1.00).	1.22 (1.05–1.43) *		1.00 (0.85–1.18)	0.013
	OR adjusted 1			1.66 (0.98–2.81)		1.40 (0.82–2.37)	0.073
Child’s zBMIscore	Crude OR		Ref. (1.00).	0.99 (0.85–1.16)		0.95 (0.81–1.11)	0.792
	OR adjusted 1			0.83 (0.50–1.37)		1.01 (0.61–1.69)	0.158
2022–2023			**No BF (** ***n*** ** = 777)**	**≤ 6 months BF** **(** ***n*** **= 923)**		**> 6 months BF** **(** ***n*** **= 1109)**	**p-value**
Primary caregiver age	Crude OR		Ref. (1.00).	1.08 (0.89–1.31)		0.95 (0.79–1.14)	0.346
	OR adjusted 1			1.65 (0.83–3.27)		1.36 (0.69–2.67)	0.143
Primary caregiver education (university or not)	Crude OR		Ref. (1.00).	0.37 (0.30–0.45) **		0.26 (0.21–0.32) **	< 0.001
	OR adjusted 1			0.67 (0.32–1.4)		0.44 (0.21–0.91) *	< 0.001
Primary caregiver employment status (working or not)	Crude OR		Ref. (1.00).	0.27 (0.22–0.33) **		0.29 (0.24–0.34) **	< 0.001
	OR adjusted 1			0.99 (0.45–2.22)		0.86 (0.39–1.90)	0.615
Child’s Weight (kg)	Crude OR		Ref. (1.00).	1.05 (0.88–1.25)		0.74 (0.63–0.88) **	< 0.001
	OR adjusted 1			0.77 (0.39–1.51)		0.55 (0.29–1.07)	0.008
Child’s Height (cm)	Crude OR		Ref. (1.00).	1.19 (1.00-1.42)		0.78 (0.67–0.93) **	< 0.001
	OR adjusted 1			0.92 (0.48–1.79)		0.63 (0.33–1.21)	0.005
Child’s zBMIscore	Crude OR		Ref. (1.00).	0.94 (0.79–1.11)		0.96 (0.82–1.14)	0.751
	OR adjusted 1			0.64 (0.33–1.24)		0.60 (0.32–1.17)	0.322

Abbreviations: BF; Breastfeeding. BMI; Body Mass Index. 95%CI: 95% Confidence Interval; OR: Odds Ratio. OR 1 was adjusted by exclusive breastfeeding; p-values: *p < 0.05 and **p < 0.01

Table 6 presents the associations between primary caregiver sociodemographic characteristics, children’s anthropometric indicators, and (BF) duration in both study periods.

Across both surveys, BF duration tended to be associated with caregiver educational level. In crude models, children breastfed for ≤ 6 months or > 6 months showed substantially lower odds of having caregivers without a university education compared with non-breastfed children. This pattern remained generally consistent after adjustment, particularly in the 2019–2020 cohort, where the odds ratios suggested a strong association between longer BF duration and higher caregiver educational attainment. Caregiver employment status showed a similar tendency in crude models in both periods, with children who had been breastfed more frequently having caregivers who were working. However, after adjustment for exclusive BF duration, these associations were attenuated and CIs were wide, suggesting considerable uncertainty in the estimates. Regarding caregiver age, the estimated associations were modest and the CIs generally included the null value in both study periods, indicating no clear relationship between caregiver age and BF duration.

For children’s anthropometric characteristics, crude models suggested some differences in weight and height according to BF duration, particularly in the 2022–2023 cohort, where longer BF tended to be associated with lower weight and height estimates. Nevertheless, these associations were attenuated after adjustment and CIs frequently overlapped the null value.

## Discussion

Consistent with our priori hypothesis, the current study suggests a potential association between BF duration during early childhood and dietary behaviors in later childhood and adolescence. Assessing two large, nationally representative studies of Spanish youth, there were consistent associations between longer BF duration and higher adherence to the MD, as well as healthier eating habits such as regular breakfast consumption and higher intake cereals at breakfast, vegetables, fruit, fish and rice/pasta. These associations were observed across both survey waves, and extend the well-documented health benefits of BF [5, 32], although their magnitude and consistency varied depending on the outcome and adjustment models. Given the observational design and the long-time interval between BF exposure and outcome assessment, BF duration should not be interpreted as a causal determinant of later dietary behaviors. Rather, it may represent an early-life marker of broader family, cultural, and socio-educational factors that influence children’s dietary patterns over time. Overall, across both study periods, longer BF duration, particularly beyond six months, showed a tendency toward healthier dietary patterns and behaviors aligned with the MD, although many associations weakened after accounting for exclusive BF duration and parental sociodemographic characteristics. Given the exploratory nature of the item-level analyses and the relatively wide CIs observed in the adjusted models, these findings should be interpreted cautiously.

In the SENDO project, a Spanish project assessing lifestyle effects of obesity in children, the effect of BF duration on MD adherence in the offspring was examined in 4 to 5-year-old preschool children, also showed a higher adherence to the MD as BF time increased [32]. They also found that longer BF duration was associated with lower consumption of ultra-processed foods in 4–5 years old [33], while our results partially align with these findings regarding MD adherence; we did not observe consistent associations with ultra-processed food consumption in older children and adolescents. In the current study, the population was expanded to include 8 to 16-year-old children and adolescents and compared data from both editions. These discrepancies may be explained by differences in age range, as dietary autonomy increases with age, potentially diluting early-life associations. It is also noteworthy that we stratified our current sample into 3 categories: non-breastfed children, children breastfed up to the minimum recommendations, and children breastfed beyond 6 months, while in the SENDO project the second category didn’t include the six-months minimum. This approach allowed us to explore whether BF, beyond the minimum recommended duration provides additional benefits in adhering to the MD and following healthier habits, seems to be the case. Methodological differences in BF categorization between studies may also contribute to the observed inconsistencies.

Evidence suggests that maternal and parental dietary patterns, exclusive BF during the first six months, and the timing of complementary food introduction may act as early-life markers associated with children’s later dietary behaviors, including adherence to the MD and potential health outcomes [34–38]. In particular, complementary feeding practices between 6 and 24 months have also been associated with later adiposity and dietary intake trajectories, suggesting that early dietary exposures beyond BF may contribute to the establishment of long-term eating behaviours [39, 40]. However, this information was not available in the present study, and therefore could not be accounted for in the analyses. This represents a potential source of residual confounding that should be acknowledged when interpreting the observed associations.

Our findings indicate that several associations between BF duration and later dietary behaviours were attenuated after adjustment for exclusive BF and parent’s education, employment status and age. Specifically, when exclusive BF during the first six months and parents’ sociodemographic were included in the models, the strength of many associations decreased or lost statistical significance. This attenuation suggests that part of the observed associations between BF duration and later dietary behaviours may be explained by broader family-level and socio-educational factors rather than BF duration alone.

By doing those adjustments, we aimed to explore whether the observed associations between BF and dietary patterns were driven by BF duration in general, or specifically by exclusive BF during the first six months, and whether parental situation could influence those associations. We chose these variables as they were considered the most relevant indicators of the early-life family context, and because our primary interest was to examine the potential effect of extended BF duration on later dietary habits. Given the strong correlation between exclusive and total BF duration, adjusting for exclusive BF constitutes a highly conservative modelling approach that may underestimate the true magnitude of the associations. We nevertheless applied this adjustment to clarify whether the observed patterns were driven specifically by exclusive BF during the first months of life or by longer BF duration overall.

Overall, the results suggest that caregiver educational level may be the sociodemographic factor most consistently associated with BF duration, whereas associations with caregiver age, employment status, and children’s anthropometric indicators were less consistent. Notably, some associations persisted even after adjustment, suggesting that BF duration may still capture aspects of early feeding practices or family environments not fully accounted for by the included covariates. However, we acknowledge that future studies could benefit from a broader set of adjustments and longitudinal designs to better explore causality and to compare these results with more fully controlled models. Furthermore, the variability observed between survey waves and across dietary outcomes underscores the complexity of these relationships and highlights the need for cautious interpretation. Differences in social context, cohort composition, and lifestyle behaviours over time may partially explain inconsistencies between the 2019–2020 and 2022–2023 waves.

Despite the well-established benefits of BF, a significant number of families in both PASOS studies (2019–2020 and 2022–2023) reported not BF their children. Previous literature identified several socio-cultural and structural barriers influencing BF practices, such as maternal employment, limited maternity leave, and societal rules [41–46]. According to the 2023 *Global Breastfeeding Scorecard* [47], exclusive BF rates remain low in upper-middle-income countries, highlighting the need for targeted strategies to promote and support BF practices. Exclusive BF during the first six months is recommended by WHO and UNICEF^1^ and was considered as cut-off point in the present study. In our studies, the mean duration of exclusive BF did not meet these recommendations, with mean durations being 3.6 months in 2019–2020 and 3.9 months in 2022–2023.

Taken together, our findings indicate that BF duration is associated with later dietary patterns during childhood and adolescence, likely reflecting early-life family-level and socio-educational contexts rather than acting as an independent determinant. The attenuation of several associations after adjustment for exclusive BF during the first six months and parent’s education, employment status and age supports this interpretation and highlights the complex interplay between early feeding practices and broader contextual factors.

By examining these associations in school-aged children and adolescents across two survey waves, this study extends previous evidence beyond early childhood and helps to clarify the nature and persistence of these relationships over time. At a conceptual level, the findings should be interpreted within a framework of early-life behavioral clustering and family-driven health trajectories, in which BF duration may function as an early-life marker of a constellation of family characteristics—such as health-related knowledge, dietary norms, and lifestyle practices—that tend to cluster and persist across childhood and adolescence.

The long temporal gap between BF exposure and dietary outcomes, while limiting causal inference, is central to the contribution of this study. Nevertheless, maternal recall of BF has been shown to be a valid and reliable measure in epidemiological research, particularly for BF initiation and overall duration, even when recalled several years after delivery. Previous validation studies have reported high agreement between maternal recall and prospectively collected data, suggesting that BF history can be reliably captured through retrospective questionnaires. Therefore, although recall bias cannot be completely ruled out, the use of maternal-reported BF in population-based studies is generally considered an acceptable and informative marker of early-life exposure [48–50].

Assessing associations beyond early childhood allows evaluation of whether early feeding-related markers retain relevance as parental control over diet decreases and children gain greater dietary autonomy. In this context, the persistence or attenuation of associations across age groups and survey waves provides insight into which dietary behaviors are more strongly embedded within early family environments, and which are more susceptible to later-life contextual influences.

### Strengths and limitations of the study

This study has several strengths. First, it is based on two large, nationally representative cohorts of Spanish children and adolescents, increasing the generalizability of the findings within this context. Second, detailed BF histories were collected, including duration and exclusiveness, allowing a nuanced analysis of their associations with later dietary habits. Third, dietary behaviors were assessed using the validated KIDMED index, providing reliable measures of adherence to the MD. Fourth, the study complements previous research on the association between BF and nutritional behaviors in younger populations in Spain, highlighting the relevance of preserving healthy dietary habits and lifestyles. Finally, adjustment for parent’s education, employment status and age allowed partial consideration of family-level confounding.

However, this study also has several limitations. Its observational design, combined with the long temporal gap between exposure and outcome, excludes causal inference; associations observed should be interpreted as potential early-life markers of broader family-level, socio-educational, and dietary environment influences rather than direct effects of BF duration. BF data were caregiver‑reported, which may be subject to recall bias. Although maternal recall of BF duration has shown good validity even many years after delivery, some degree of misclassification cannot be excluded. Such misclassification, whether non‑differential or potentially differential, should be considered when interpreting the strength of the observed associations. Although we adjusted for key covariates, including exclusive BF during the first six months and parental education, employment status and age, residual confounding remains a key issue, as unmeasured factors could contribute to the observed associations. Because exclusive BF duration is closely related to total BF duration, adjusting for exclusive BF may represent an over adjustment that could attenuate the associations observed. This should be considered when interpreting the results. Differences between survey waves and age groups further highlight the heterogeneity and complexity of these relationships, emphasizing the need for cautious interpretation. Finally, as the study population is living in a Mediterranean context, the dietary behaviors captured by the KIDMED index may not be directly comparable to populations outside Mediterranean settings, which limits generalizability.

Future studies, ideally longitudinal, are needed to clarify the persistence and potential causal mechanisms underlying these associations.

## Conclusions

The current findings suggest that longer BF duration is associated with healthier dietary habits and greater adherence to the MD in children and adolescents. These associations should be interpreted as reflecting early-life markers of family, cultural, and socio-educational influences, rather than independent or causal effects. The attenuation observed after adjustment for exclusive BF during the first six months and parent’s education, employment status and age further highlights the potential role of early feeding practices and broader family-level factors in shaping children’s dietary behaviors. Overall, this study provides an observational perspective on the relationship between BF duration and later dietary patterns, offering insights that may be informative for future research and public health monitoring.

## Data Availability

There are restrictions on the availability of data for this trial due to the signed consent agreements around data sharing, which only allow for access to external researchers for studies following the project purposes. Requestors wishing to access the trial data used in this study can make a request to pep.tur@uib.es.

## Abbreviations

ANOVA: Analysis of Variance
BMI: Body Mass Index
BF: Breastfeeding
KIDMED: Index of adherence to the Mediterranean Diet in children
MD: Mediterranean Diet
OR: Odds Ratio
PASOS: The Physical Activity, Sedentarism, and Obesity in Spanish Youth study
SD: Standard deviation
SENDO: The Child Monitoring for Optimal Development study
UNICEF: United Nations Children’s Fund
WHO: World Health Organization
